# Actin Recruitment to the *Chlamydia* Inclusion Is Spatiotemporally Regulated by a Mechanism That Requires Host and Bacterial Factors

**DOI:** 10.1371/journal.pone.0046949

**Published:** 2012-10-11

**Authors:** Elizabeth Chin, Kelly Kirker, Meghan Zuck, Garth James, Kevin Hybiske

**Affiliations:** 1 Division of Infectious Diseases, School of Public Health, University of California at Berkeley, California, United States of America; 2 Center for Biofilm Engineering, Montana State University, Bozeman, Montana, United States of America; University of Würzburg, Germany

## Abstract

The ability to exit host cells at the end of their developmental growth is a critical step for the intracellular bacterium *Chlamydia*. One exit strategy, extrusion, is mediated by host signaling pathways involved with actin polymerization. Here, we show that actin is recruited to the chlamydial inclusion as a late event, occurring after 20 hours post-infection (hpi) and only within a subpopulation of cells. This event increases significantly in prevalence and extent from 20 to 68 hpi, and actin coats strongly correlated with extrusions. In contrast to what has been reported for other intracellular pathogens, actin nucleation on *Chlamydia* inclusions did not ‘flash’, but rather exhibited moderate depolymerization dynamics. By using small molecule agents to selectively disrupt host signaling pathways involved with actin nucleation, modulate actin polymerization dynamics and also to disable the synthesis and secretion of chlamydial proteins, we further show that host and bacterial proteins are required for actin coat formation. Transient disruption of either host or bacterial signaling pathways resulted in rapid loss of coats in all infected cells and a reduction in extrusion formation. Inhibition of *Chlamydia* type III secretion also resulted in rapid loss of actin association on inclusions, thus implicating chlamydial effector proteins(s) as being central factors for engaging with host actin nucleating factors, such as formins. In conclusion, our data illuminate the host and bacterial driven process by which a dense actin matrix is dynamically nucleated and maintained on the *Chlamydia* inclusion. This late stage event is not ubiquitous for all infected cells in a population, and escalates in prevalence and extent throughout the developmental cycle of *Chlamydia*, culminating with their exit from the host cell by extrusion. The initiation of actin recruitment by *Chlamydia* appears to be novel, and may serve as an upstream determinant of the extrusion mechanism.

## Introduction

*Chlamydia* spp. continue to have a major burden on global public health. *C. trachomatis* is the leading cause of sexually transmitted infection, responsible for an estimated 90 million new cases annually worldwide [1], and is also the primary etiologic agent of the blinding disease trachoma [2]. The annual incidence rate of trachoma is 80 million worldwide [3]. Furthermore, *Chlamydia* infections can lead to ectopic pregnancy and pelvic inflammatory disease [2], [4], [5], enhance HIV transmission [6], and may be a positive risk factor for atherosclerosis and cervical cancer [7], [8]. Chlamydiae are obligate intracellular bacteria that are characterized by a biphasic developmental cycle [9]. Infections in the host begin with contact of *Chlamydia* elementary bodies (EB), the infectious and metabolically inert form of the bacteria, with columnar epithelial cells. *Chlamydia* EB attach to and internalize into epithelial cells, and take residence in a vacuole known as the inclusion. Within this protective intracellular niche, *Chlamydia* convert into the larger, metabolically active reticulate bodies (RB) and undergo successive rounds of replication and division. This proceeds until the bacteria number in the hundreds and the vacuole has swollen to fill the host cell; during this time *Chlamydia* asynchronously convert back into EB and are released from the host cell.

In accordance with the fundamental importance of exiting host cells at the end of their intracellular growth, *Chlamydia* have evolved two non-redundant strategies for accomplishing this task [10]. The first, extrusion, is a packaged release of bacteria in which the vacuole pinches off and exits the cell within a membrane-encased compartment; this leaves the original host cell intact, often with a residual chlamydial inclusion. Lysis, the second exit pathway, is a destructive process that is mediated by cysteine proteases and the sequential rupture of vacuole, nuclear and plasma membranes, culminating in the release of free bacteria.

Extrusion is critically dependent on actin polymerization and cellular pathways that regulate actin dynamics, including N-WASP [10]. Based on these molecular requirements for *Chlamydia* exit, it is likely that these pathways are engaged by *Chlamydia* from within the inclusion, or from the inclusion membrane itself. This hypothesis is consistent with the emerging theme that the inclusion represents the keystone of *Chlamydia* pathogenesis. *Chlamydia* uniquely modify and interact with this compartment in order to avoid endolysosomal trafficking pathways, acquire lipids from the host cell, activate Src family kinases, induce cytoskeletal rearrangements and limit detection by immune surveillance pathways [11]–[17].

The ability of *Chlamydia* to secrete effector proteins by type III secretion (TTS) and manipulate actin at early steps of infection is well established. The *Chlamydia* effector protein CT456 (TARP) is secreted during bacteria entry into host cells and initiates actin polymerization at attachment foci by a mechanism that involves Rac, WAVE2 and Arp2/3 [18]–[22]. The recently described *Chlamydia* effector protein CT166 may also play a role in actin nucleation during bacteria entry [23]. Actin and the intermediate filament proteins vimentin, keratin-8 and keratin-18 have been shown to accumulate on *Chlamydia* inclusions [17], [24]; however, since these phenomena are reported for early times of infection [17], [24], they are unlikely to play a significant role in the actin rearrangements that mediate extrusion, which occurs nearly 40 hours later [10].

Based on preliminary data, we hypothesized that at late times of infection, *Chlamydia* direct the recruitment of actin to the mature inclusion membrane as an initial step in the extrusion exit pathway. We report that actin structures are dynamically recruited to the *Chlamydia* inclusion beginning at 20 hpi for *C. trachomatis*, and the prevalence of this event increases throughout the chlamydial developmental cycle. Importantly, this mechanism requires both host signaling pathways associated with actin polymerization and *Chlamydia* type III secretion products. Actin coat formation is critical for extrusion formation.

## Results

### Actin recruitment to the chlamydial inclusion over the developmental cycle

Actin polymerization and nucleation factors have been shown to mediate cellular release of *Chlamydia* spp. by the extrusion mechanism [10], and actin recruitment to the *Chlamydia* vacuole (inclusion) has been reported [24]. Since the engagement of actin signaling networks is the first discernible step in extrusion [10], we hypothesize that it is due to bacterial factors that are either secreted into the host cytoplasm or are inserted into the inclusion membrane. To address this, we probed for actin recruitment in *Chlamydia*-infected HeLa cells using the LifeAct-TagGFP reporter (subsequently called actin-GFP), which consists of a 17 amino acid peptide that binds actin, fused to TagGFP [25]. LifeAct is a valuable tool that does not interfere with actin dynamics, accurately labels actin structures in living cells and enables the rapid detection and characterization of actin morphology in real-time [25]. LifeAct has been used previously to illuminate actin polymerization on *Cryptococcus* phagosomes [26].

**Figure 1:**
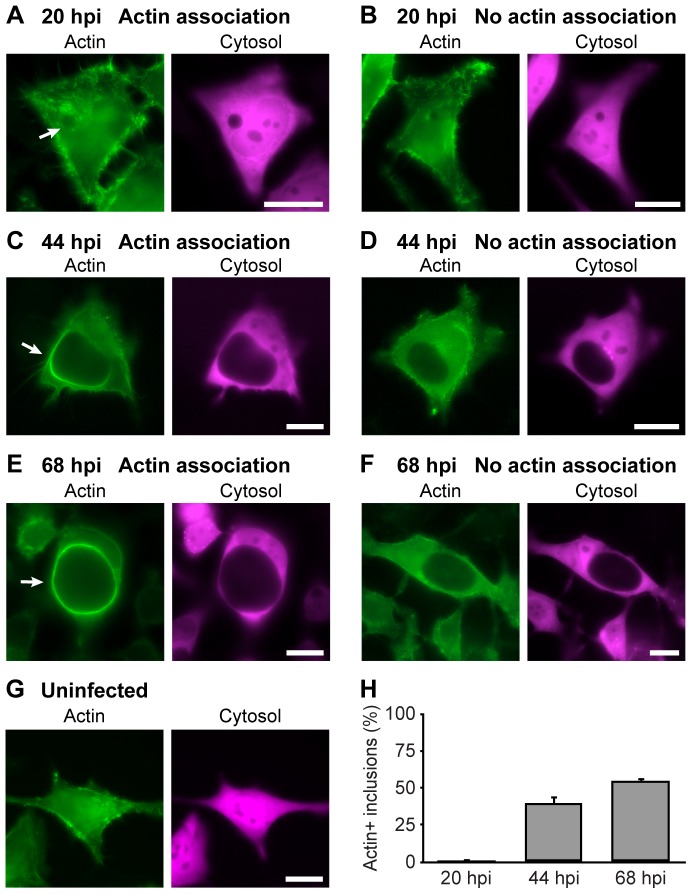
Assembly of actin coats on *Chlamydia* inclusions increases in strength and prevalence over the developmental cycle. Cellular actin in HeLa cells was labeled with LifeAct-GFP (green) and cytosolic DsRed (magenta). Cells were infected with *C. trachomatis* and imaged live at 20 hpi (**A,B**), 44 hpi (**C,D**), 68 hpi (**E,F**) or uninfected (**G**). Examples of inclusions with actin association (**A,C,E**) and no recruitment (**B,D,F**) are provided. The example in (**A**) depicts actin clouds but not coat formation. Inclusions with actin recruitment (**A,C,E**) are marked with arrows. Scale bars = 20 µm. (**H**) Quantitation of inclusions with actin coats, at indicated times hpi. Error bars denote the SEM, >1200 inclusions were scored from a total of n≥5 experiments.

HeLa cells were co-transfected with cytosolic DsRed—to enable detailed visualization of *Chlamydia* inclusions [10]—and actin-GFP, and infected with *C. trachomatis* LGV serovar L2. *Chlamydia* inclusions were readily detected in tandem with actin-GFP by live cell fluorescence microscopy. Representative examples are illustrated in Figure 1. Infected cells at 20 hpi exhibited little to no significant association between actin and the bacterial inclusion (Figure 1B). Actin morphology in these cells was largely indistinguishable from that of uninfected cells (Figure 1G). On occasion, actin ‘clouds’—regions of non-filamentous actin with moderate intensity in close proximity to the inclusion—were detected (Figure 1A). By 44 hpi many cells were found with strong actin recruitment to the cytosolic surface of the inclusion (Figure 1C), although only for a subset of cells (Figure 1D). For those cells with redistributed actin, actin-GFP was shown to partially coat the inclusion, and staining was most pronounced on regions of the inclusion distal to the nucleus. At 68 hpi, significantly more cells in a given population were found with strong staining of actin, and recruitment was typically thicker with fully-formed complete ‘actin rings’ around inclusions (Figure 1E). The three dimensional spatial distribution of actin-GFP cages and endogenous actin was additionally examined (Figure S1, S2, Video S1, Video S2).

The extent of actin recruitment to *Chlamydia* inclusions was quantitatively measured by enumerating inclusions that were positive for significant actin recruitment. The data revealed a dramatic increase in the prevalence of inclusions with actin recruitment over the developmental cycle (Figure 1H).

### Ultrastructural characterization of actin cytoskeleton in Chlamydia-infected cells

We next sought to characterize the precise ultrastructural details of actin deposition on the *Chlamydia* inclusion. Fluorescence microscopy based approaches are insufficient for informing important details in actin structures. This, coupled with the small diameter of actin filaments, necessitates the use of electron microscopy for the resolution of actin cytoskeletal structure. We subjected infected cells to detergent extraction scanning electron microscopy (SEM), an established method for dissolving membrane bilayers while preserving cytoskeletal structures [27], [28]. The importance of this approach for intracellular pathogens like *Chlamydia* is that it enables resolution of the respective spatial distributions of bacteria and actin structures with inclusion and plasma membranes removed.

**Figure 2:**
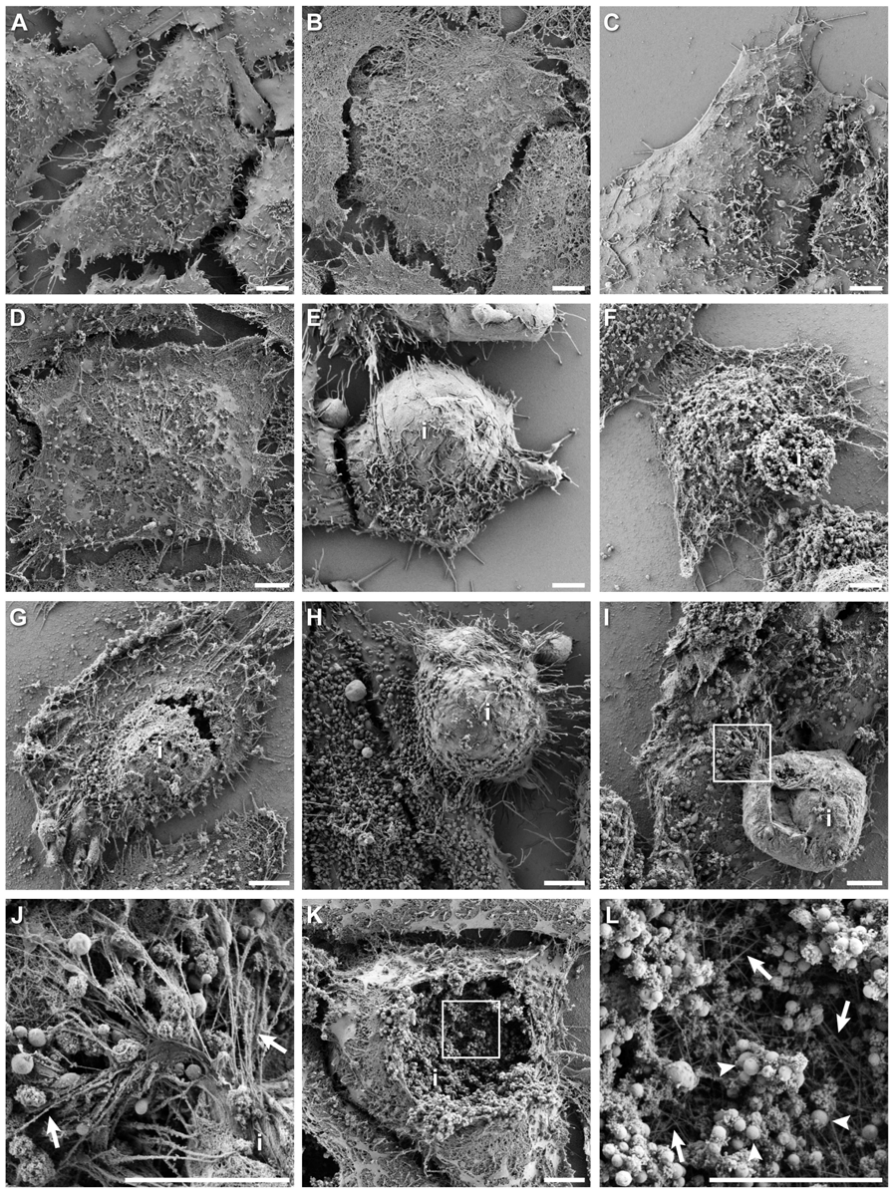
High resolution actin cytoskeleton ultrastructure in *Chlamydia*-infected cells by detergent extraction scanning electron microscopy. (A) Uninfected cell processed without detergent extraction, showing membrane topology. (B) Uninfected cell with membranes removed and actin cytoskeleton preserved; cytoskeletal matrix was revealed. (C) *Chlamydia* infected cell at 20 hpi without detergent extraction, membranes were intact and appearance is indistinguishable from uninfected cells. (D) *Chlamydia* infected cell at 20 hpi with membranes extracted and actin cytoskeleton preserved; small inclusions and/or bacteria were difficult to identify at this time. (E) *Chlamydia* infected cell at 44 hpi with membranes intact; underlying large inclusion *i* is evident in this example, and plasma membrane on protruding surface was smoother with reduced microvilli density. (F) *Chlamydia* infected cell at 44 hpi with membranes removed and cytoskeleton preserved; in this example, little or no actin was recruited to the inclusion, which is readily visible as a spheroid structure of packed bacteria. (G) Detergent-extracted, *Chlamydia* infected cell at 44 hpi exhibiting significant actin polymerization on the inclusion; inclusion associated actin is continuous with actin matrix of the cell body. (H) *Chlamydia* infected cell at 68 hpi with membranes intact, showing large protrusion of underlying inclusion. (I) Detergent-extracted, *Chlamydia* infected cell at 68 hpi showing large inclusion densely wrapped with actin. Boxed region is enlarged in (J), and illustrates the fibrous actin connections between large inclusion, or possibly an extrusion, with the cytoskeletal matrix of the cell. Numerous actin bundles were observed, some are marked by arrows. (K) A ruptured *Chlamydia* infected cell at 68 hpi with membranes removed and actin structures preserved. Dense layers of actin on the inclusion were seen, and contents of inclusion are revealed to contain hundreds of individual bacteria. Boxed region is enlarged in (L) to highlight the network of actin filaments (arrows) effacing the inclusion surface and show individual bacteria (arrowheads). Scale bars = 5 µm.

At specific times after infection with *C. trachomatis*, cells were processed and imaged by SEM. In uninfected cells, this technique revealed cells with their plasma membranes removed, unmasking a dense network of actin in the cell body and fibrous structures at the cell periphery (Figure 2B). This was in contrast to cells that were imaged by SEM without detergent extraction (Figure 2A), wherein plasma membranes were preserved with intact surface details such as microvilli.

Infected cells at 20 hpi (Figure 2C) were mostly indistinguishable from uninfected cells, even for samples that were detergent extracted (Figure 2D). At these early times, bacterial loads inside cells were too low and the cytoskeletal network of the host cell was too dense to allow for identification of dissolved inclusions and individual bacteria.

By 44 hpi, infected cells were much larger and frequently observed with bulbous surfaces (see example in Figure 2E). The swollen regions, under which inclusions were presumed to reside, were markedly reduced in microvillus density, consistent with previous reports [29]. Detergent extracted (membranes stripped) cells at 44 hpi yielded two predominant outcomes: inclusions with little or no actin recruitment (Figure 2F), and inclusions with significant layers of actin networks coating their surfaces and connected to cell body actin (Figure 2G), consistent with prior data.

Cells at 68 hpi were distended and clearly shown to harbor large inclusions (Figure 2H); actin ultrastructural details on and around the inclusion was markedly different than for earlier times. Actin was densely layered on the surface of the inclusion and precluded observation of any bacteria (Figure 2I). Frequently, the actin coat exhibited folding or inward invagination into the inclusion (Figure 2I) in a manner that is consistent with previously reported findings [10]. The extent of actin deposition on the inclusion was surprisingly dense and made resolution of actin structural details, for example branching or fibrous characteristics, difficult. However, some information was obtained from regions where the inclusion-associated actin interacted with the actin network of the cell body. In these defined areas, bundles of actin connecting the inclusion to the cell body were frequently observed (Figure 2J). In another example, the inclusion had been ruptured open, enabling resolution of bacteria inside the inclusion (Figure 2K–L), the network of actin behind the inclusion (Figure 2L) and a cross-sectional view of the thickness of the actin coat surrounding the inclusion (Figure 2K).

### Differences in actin recruitment among chlamydiae

**Figure 3:**
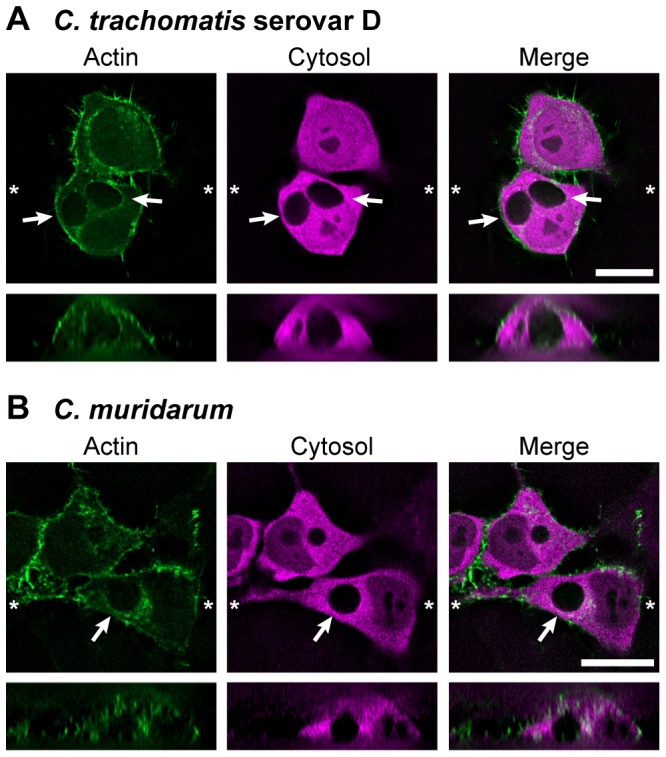
Actin distribution in cells infected with *C. trachomatis* serovar D and *C. muridarum*. HeLa cells transfected with LifeAct-GFP (green) and cytosolic DsRed (red) were infected with *C. trachomatis* serovar D (**A**) or *C. muridarum* (**B**) and imaged by live fluorescence microscopy at 20 hpi for *C. trachomatis* serovar D and 44 hpi for *C. muridarum*. Over 35 z-stacks were acquired at 0.5 µm intervals. Image stacks were additionally processed by deconvolution. Representative fields of cells for each time point are depicted. Asterisks (*) mark the locations where orthogonal planes in *xz* were taken. Arrows mark the inclusions. Scale bars = 20 µm.

The conservation of this phenomenon for chlamydiae was tested using *C. trachomatis* serovar D, *C. muridarum* and *C. caviae* GPIC. Inclusions containing *C. trachomatis* serovar D (Figure 3A) or *C. caviae* GPIC (not shown) recruited actin, although not to the same extent as for *C. trachomatis* L2. *C. muridarum* inclusions exhibited only minimal actin recruitment (Figure 3B). Arcs or ring-like structures of actin were never detected on *C. muridarum* inclusions, and the only manner of association was punctate regions of actin staining in proximity to and on the surface of these inclusions (Figure 3B). In this regard, the results mirror the characteristics of microdomains of Inc proteins and active Src family kinases that have been recently reported [16], [30]. For all additional chlamydial strains tested, the weaker staining pattern of actin precluded our ability to robustly quantitate the event as a function of time.

To demonstrate that actin recruitment to the *Chlamydia* inclusion was not a phenomenon unique to HeLa cells, we tested its reproducibility in additional host cells. Murine L929 cells and McCoy cells were infected with *C. trachomatis* L2 and examined for their capacity to support actin recruitment to the inclusion. Both of these cells exhibited actin coats on inclusions to a similar extent, prevalence and timing as was found in HeLa cells (data not shown).

### Live actin dynamics in Chlamydia infected cells

The dynamic spatial and temporal recruitment of actin to the *Chlamydia* inclusion may be similar to the rapid, transient actin ‘flashes’ that have been described for other pathogenic vacuoles and intracellular bacteria [26], [31], [32]. Alternatively, it could be due to a slow and steady accumulation of actin on the inclusion surface. To investigate the live, temporal dynamics of actin coats on *Chlamydia* inclusions, cells at 44 hpi were mounted on a fluorescence microscope for live cell time-lapse imaging. The data revealed that actin complexes on inclusions were not stable structures, nor did they exhibit rapid flashing. The average duration of actin association on inclusions at 44 hpi was 45 minutes (see Video S3), and we were unable to successfully capture events of de novo actin coat formation. Infected cells at 20 hpi were also examined, and revealed small foci and dense pockets of actin-GFP that were frequently adjacent to inclusions and highly dynamic in character (see Video S4).

### Role of host signaling pathways in actin coat formation

We rationalized that the mechanism for actin recruitment to the *Chlamydia* inclusion would be: (*i*) entirely due to chlamydial effector proteins secreted onto the inclusion membrane and/or into the host cytosol; (*ii*) exclusively host driven; or (*iii*) an outcome of chlamydial and host factors working together. Previous data support the notion that host factors are involved in this phenomenon [24], and also that *Chlamydia* secretes proteins that interact with actin polymerization pathways at early steps of infection [18]–[22]. Our hypothesis was that the mechanism for actin recruitment to the inclusion would be complex, involving numerous host signaling pathways, and likely differentially regulated over the chlamydial developmental cycle—for example, as *Chlamydia* potentially secrete different effector proteins into the host cytosol, or as the vacuole is modified differently over time.

Our experimental design overall was to quantify the effects of host and bacterial protein perturbation on actin cage formation using actin-GFP in living cells. The most direct approach was by short-term treatment of cells with specific inhibitors of discrete cellular targets [33]–[39]. We considered and attempted genetic-based disruptions; however, these experiments are confounded by the probable and known roles of actin-associated signaling in upstream events in *Chlamydia* infection, such as entry [18]–[22]. Because of these key limitations, small molecule and peptide inhibitors were preferred for their ability to target specific times in the infectious process (i.e., 44 and 68 hpi), and additionally act on all cells in a population.

**Figure 4:**
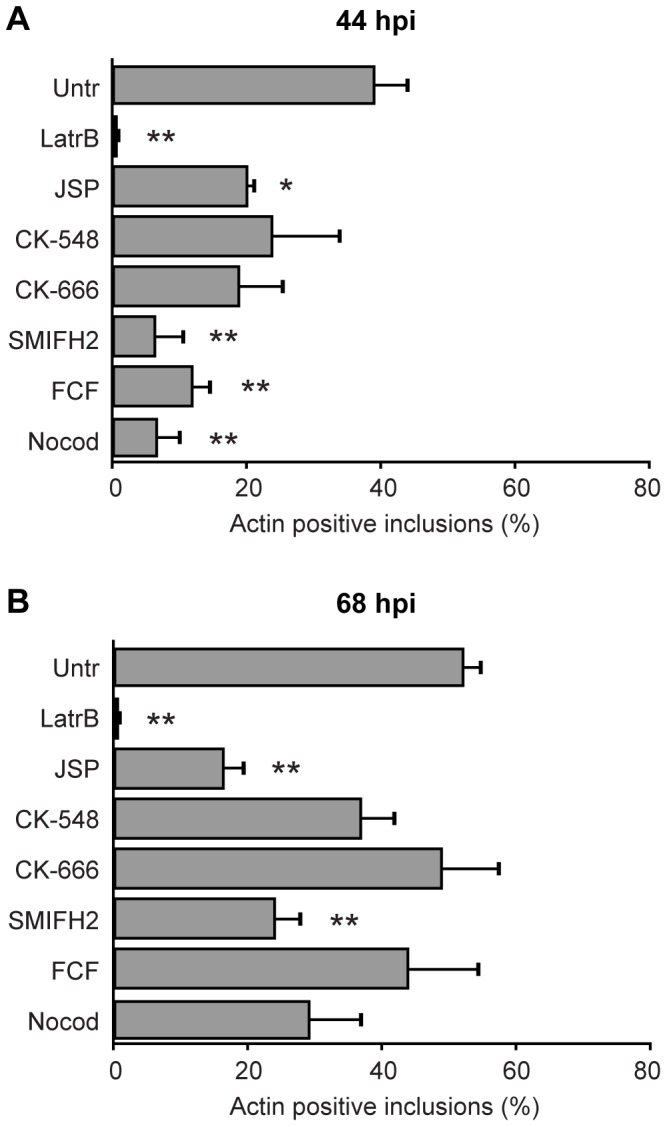
Involvement of cytoskeletal signaling pathways in actin recruitment to the *Chlamydia* inclusion. HeLa cells were infected with *C. trachomatis* and at 44 hpi (**A**) or 68 hpi (**B**) were treated for 4 h at 37°C with inhibitors targeting: actin polymerization (latrunculin B, jasplakinolide), Arp2/3 complex (CK-548, CK-666), formins (formin inhibitor SMIFH2), septins (FCF), microtubules (nocodazole), or left untreated. The numbers of inclusions with positive actin recruitment on their membranes were determined for each treatment. Error bars denote the SEM, >1000 inclusions were scored from a total of n = 4 experiments. * p<0.01, ** p<0.001.

We first investigated whether the mechanism of actin nucleation on *C. trachomatis* inclusions was mediated by Arp2/3 or formins; polymerization by these mechanisms typically result in *y*-branched or unbranched filamentous actin structures, respectively [40]. Disruption of the Arp2/3 complex had no effect on actin cage recruitment at 44 and 68 hpi, whereas formin inhibition led to a rapid decrease in the number of inclusions with actin coats at both time points (Figure 4). Prevention of actin assembly dynamics by latrunculin B or jasplakinolide similarly resulted in large reductions of inclusions with actin coats (Figure 4). Treating cells with nocodazole, to disrupt microtubules, significantly reduced actin cage prevalence at 44 but not 68 hpi (Figure 4).

The potential role of septins was examined. Septins are GTP-binding proteins that assemble into filaments and can interact with actin and other cytoskeletal proteins, and are known to mediate a wide range of structural processes in mammalian cells, including cytoskeletal dynamics and cytokinesis [41], [42]. Recently, they have been shown to interact with and form cages around intracellular bacteria [43]–[45], and a potent inhibitor of septins—forchlorfenuron (FCF)—has been characterized [35], [36], [44]. The data revealed that septins played an important role in actin cage formation and/or maintenance on *Chlamydia* inclusions at 44 hpi (Figure 4A), but had no function at 68 hpi (Figure 4B).

**Figure 5:**
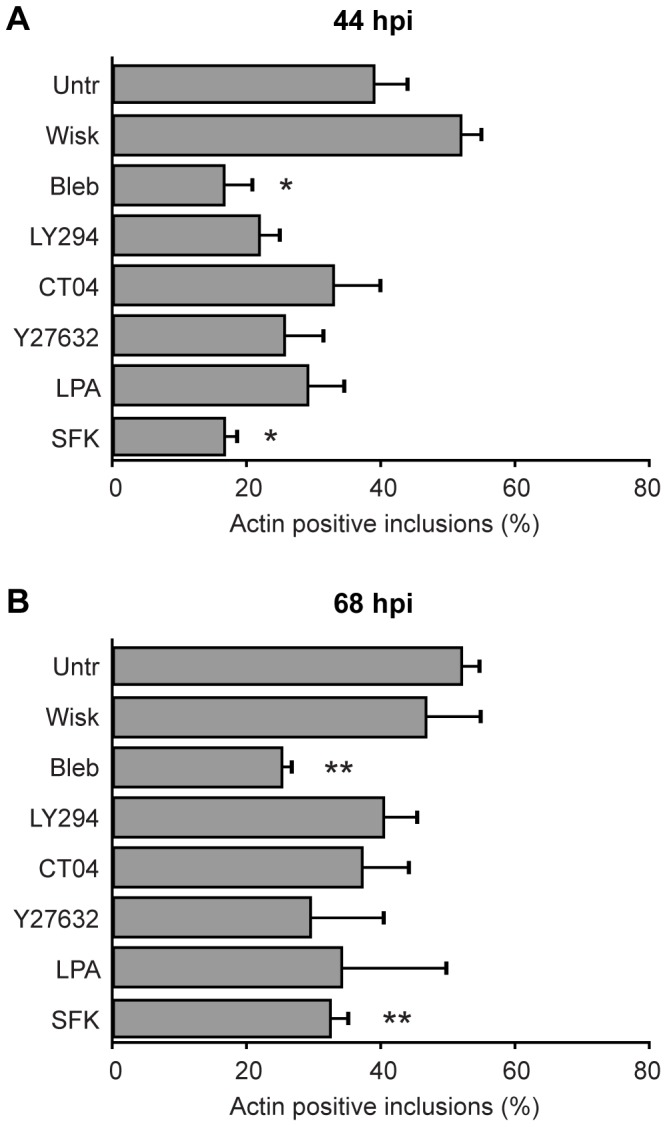
Involvement of actin-associated and additional host signaling pathways in actin recruitment to the *Chlamydia* inclusion. HeLa cells were infected with *C. trachomatis* and at 44 hpi (A) or 68 hpi (B) were treated for 4 h at 37°C with specific inhibitors against: N-WASP (wiskostatin), myosin II (blebbistatin), PI3K (LY294002), Rho GTPase (CT04), ROCK (Y27632), RhoA activator (LPA), Src family kinases (Src kinase inhibitor I), or left untreated. The numbers of inclusions with positive actin recruitment on their membranes were determined for each treatment. Error bars denote the SEM, >1000 inclusions were scored from a total of n = 4 experiments. * p<0.01, ** p<0.001.

The critical involvement of actin-associated signaling pathways in this specific process was determined by probing for the participation of N-WASP, myosin II, phosphoinositide 3-kinase (PI3K), Rho GTPases, Rho kinase (ROCK) and Src family kinases (SFK). Of these signaling pathways, only myosin II and SFK played significant roles in actin recruitment to chlamydial inclusions (Figure 5). Collectively, the data indicate that actin nucleation is formin-based and involves septins, myosin II, SFK signaling and partially requires microtubules (summarized in Table 1). Interestingly, the participation of specific signaling intermediates changed over time—for example, septins and microtubules—and therefore strengthens our hypothesis that cytoskeletal interactions with the inclusion are dynamic.

**Table 1:** Summary of reductions on percentage of actin-associated inclusions.

Target	Treatment	44 hpi	68 hpi
Host cell – significant reduction			
Actin	Latrunculin B	98	98
Actin	Jasplakinolide	48	68
Formins	SMIFH2	83	54
Myosin II	Blebbistatin	57	51
Src family kinases	Src kinase inhibitor I	57	37
Host cell – significant reduction at 44 hpi			
Septins	Forchlorfenuron	69	16 (NS)
Microtubules	Nocodazole	82	44 (NS)
Host cell – no significant reduction			
Arp2/3	CK-548	39 (NS)	29 (NS)
Arp2/3	CK-666	51 (NS)	6 (NS)
N-WASP	Wiskostatin	−33 (NS)	10 (NS)
Rho GTPase	CT04 C3 transferase	15 (NS)	28 (NS)
Rho GTPase	LPA	25 (NS)	34 (NS)
ROCK	Y27632	34 (NS)	43 (NS)
PI3K	LY294002	43 (NS)	22 (NS)
Chlamydia			
Translation	Chloramphenicol	85	n/a
Transcription	Rifampicin	85	n/a
Type III secretion	C1 compound	66	n/a

**Figure 6:**
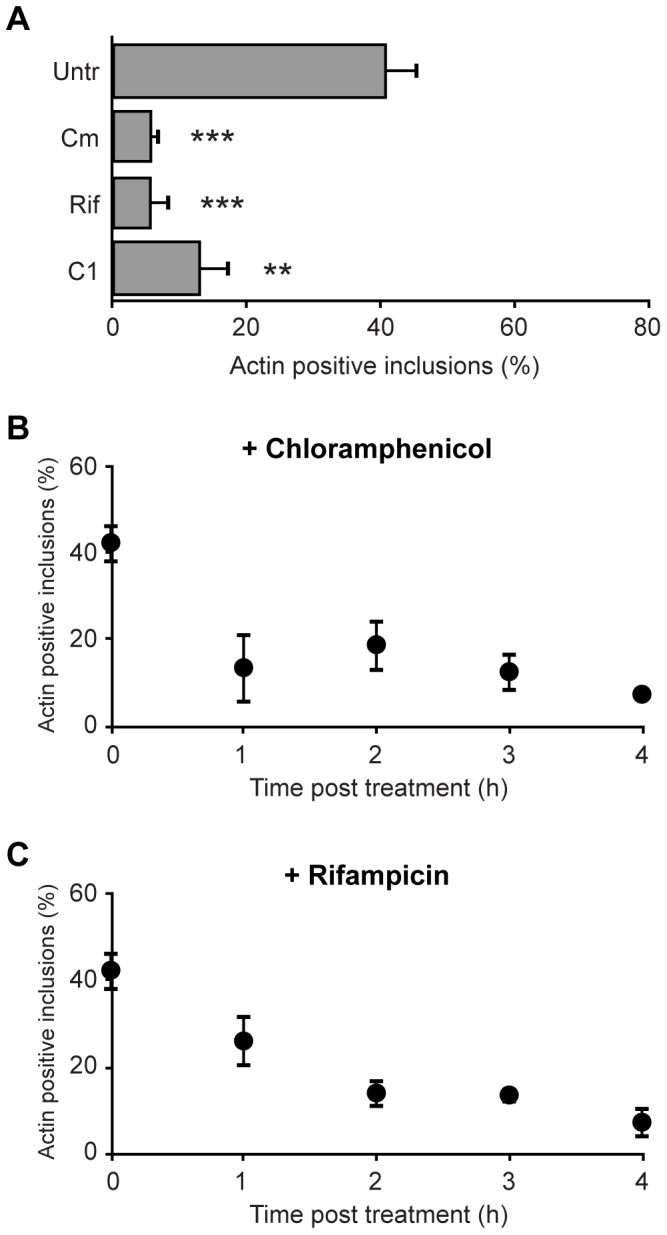
Important role for *Chlamydia* proteins in mediating actin coat formation. (**A**) HeLa cells expressing LifeAct-GFP and cytosolic DsRed were infected with *C. trachomatis* and at 44 hpi were treated for 4 h at 37°C with chloramphenicol, rifampicin, the type III secretion inhibitor C1, or left untreated. The numbers of inclusions with positive actin recruitment on their membranes were determined for each treatment. The effect of shorter treatments with chloramphenicol (**B**) or rifampicin (**C**) was determined by incubating infected cells with these compounds for times indicated. Error bars denote the SEM, >1000 inclusions were scored from a total of n = 3 experiments. ** p<0.001 *** p<0.0001.

Summary data were taken from Figures 4, 5 and 6, and represent the percent reductions in the number of actin-associated inclusions upon inhibitory treatment, compared to untreated controls. NS signifies values that were not statistically significant, p<0.01.

### Role of chlamydial proteins in actin coat formation

Chlamydiae can activate actin polymerization in host cells by at least one effector protein that is secreted into the host cytosol during entry [18]–[23]. Therefore, we hypothesized that intravacuolar *Chlamydia* might also play a central role in initiating actin polymerization on the inclusion membrane surface, and we tested this by treating cells for 4 h at 44 hpi with chloramphenicol, rifampicin or the type III secretion (TTS) inhibitor C1 compound [46]–[47]. All three treatments resulted in a dramatic decrease in the number of actin positive inclusions (Figure 6A, Table 1). Overnight treatment with all three compounds resulted in similar disruptive effects (data not shown).

We conducted a time analysis of chloramphenicol and rifampicin treatment and determined that as little as one hour treatment of infected cells with chloramphenicol was sufficient to disrupt the actin association phenotype (Figure 6B); lengthier treatment times did not meaningfully decrease the number of actin positive cells further. Similar kinetics were obtained for rifampicin treatment, although with a small delay that was consistent with its mechanism of action (Figure 6C). These data demonstrate the essential role of chlamydial protein(s) in directing the recruitment of actin on the inclusion surface, and, furthermore, that these factor(s) have a short half-life inside the infected host cell. These dynamics are in agreement with live imaging results and suggest that the expression and type III secretion of as-yet undetermined *Chlamydia* protein(s) are necessary to initiate and maintain actin polymerization on the inclusion.

**Figure 7:**
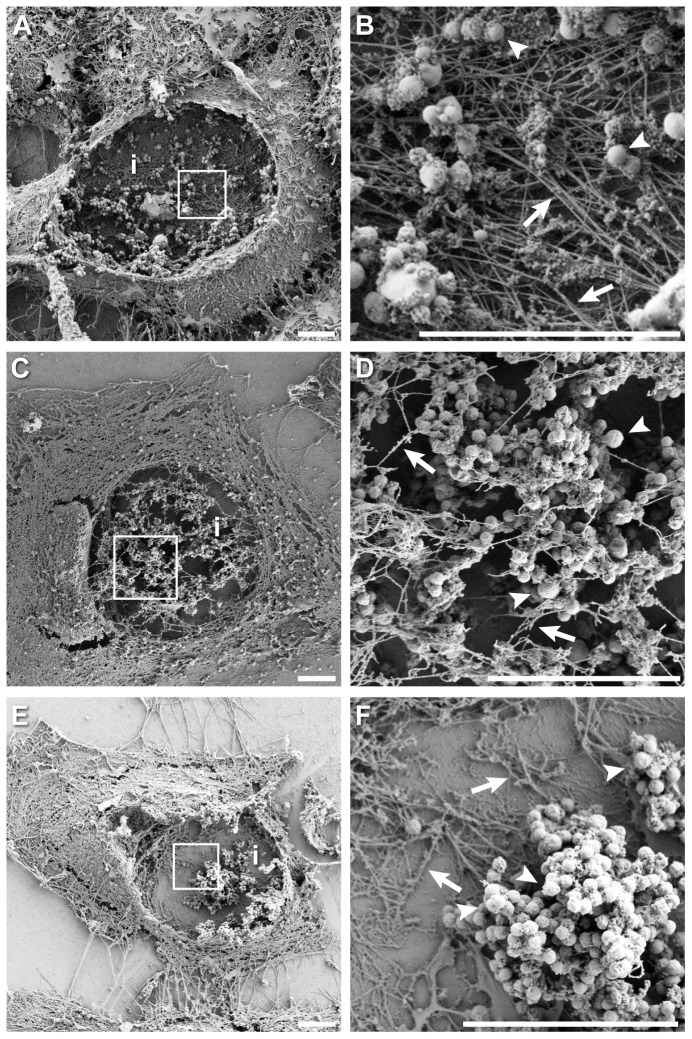
Effect of chloramphenicol and rifampicin treatment on actin ultrastructure in *Chlamydia* infected cells. (**A**) *Chlamydia* infected cell at 44 hpi with membranes removed and actin cytoskeleton preserved. Inclusion and cell were ruptured, thereby revealing actin structures on back face of the inclusion. (**B**) Enlargement of boxed region in (**A**), showing structural details of actin network behind the dissolved inclusion surface and individual bacteria. (**C**) Detergent extracted, *Chlamydia* infected cell treated with chloramphenicol for 4 h at 44 hpi. (**D**) Enlargement of boxed region in (**C**), illustrating a reduction in the number and density of actin filaments associated with the dissolved inclusion surface; in this example actin structures were in the foreground, where inclusion membrane was previously located. (**E**) Detergent extracted, *Chlamydia* infected cell treated with rifampicin for 4 h at 44 hpi. (**F**) Enlargement of boxed region in (**E**), showing significant reduction in number and density of actin filaments on the dissolved inclusion membrane, behind where the inclusion membrane would have been. Inclusions are marked with *i*, some examples of bacteria (arrowheads) and actin filaments (arrows) are also labeled. Scale bars = 5 µm.

Finally, we performed detergent extraction SEM on chloramphenicol- or rifampicin-treated *Chlamydia*-infected cells to determine the resultant changes in actin ultrastructure on the inclusion. Cells that were treated with chloramphenicol (Figure 7C–D) or rifampicin (Figure 7E–F) revealed a dramatic reduction in association of cytoskeletal structures with chlamydial inclusions, compared to what was routinely observed for untreated cells (Figure 7A–B). These structures were presumed to be actin due to the fact that microtubule preserving agents were omitted from the extraction buffer, and structures closely resembled published data of defined actin structures [27], [28]. The effects of inhibition were most evident in regions behind *Chlamydia* inclusions, wherein actin structures appeared as thin, sparse networks (Figure 7C–F).

### Functional role of actin formation in Chlamydia extrusion

**Figure 8:**
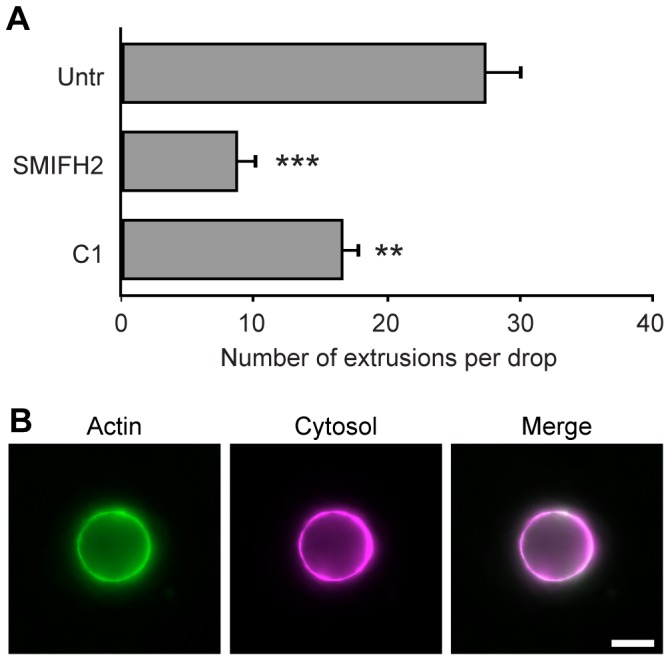
Actin coats lead to formation of *Chlamydia* extrusions. (**A**) Reduction in the number of endogenous extrusions released by cells after 4 h treatment with formin (SMIFH2) or type III secretion (C1) inhibitors, or left untreated. (**B**) Representative extrusion containing *C. trachomatis* from HeLa cells expressing LifeAct-GFP (green) and cytosolic DsRed (magenta), illustrating extensive actin coating on underlying inclusion. Error bars denote the SEM, >1000 extrusions were scored from a total of n = 3 experiments. ** p<0.001 *** p<0.0001. Scale bar = 10 µm.

The high prevalence of dense actin coats on inclusions late in the chlamydial developmental cycle suggested they played key roles for late stage events, such as *Chlamydia* exit. We therefore determined whether actin coat disruption negatively affected the downstream ability of *Chlamydia* to extrude from host cells by adapting a new approach for isolating and enumerating released extrusions from infected monolayers. Infected cells at 68 hpi were treated for 4 h with either formin or type III secretion inhibitors, or left untreated, after which endogenously released extrusions were isolated and counted on a fluorescence microscope. As shown in Figure 8A, both treatments dramatically reduced the number of extrusions produced and released from cells, with formin inhibition yielding the strongest effect. These results establish a key functional connection between actin coat formation and extrusion of chlamydial inclusions.

Nascent and released extrusions from actin-GFP cells were also isolated and visualized by fluorescence microscopy (Figure 8B), and were found to exhibit a very high association of actin recruitment with their inclusions, at 94%. This represented the largest degree of actin coating for all inclusions that were tested. Actin-GFP morphology was not simply a byproduct of the limited cytosolic space of extrusions, since for some extrusions actin-GFP was not polymerized into bright rings or coats (not shown).

**Figure 9:**
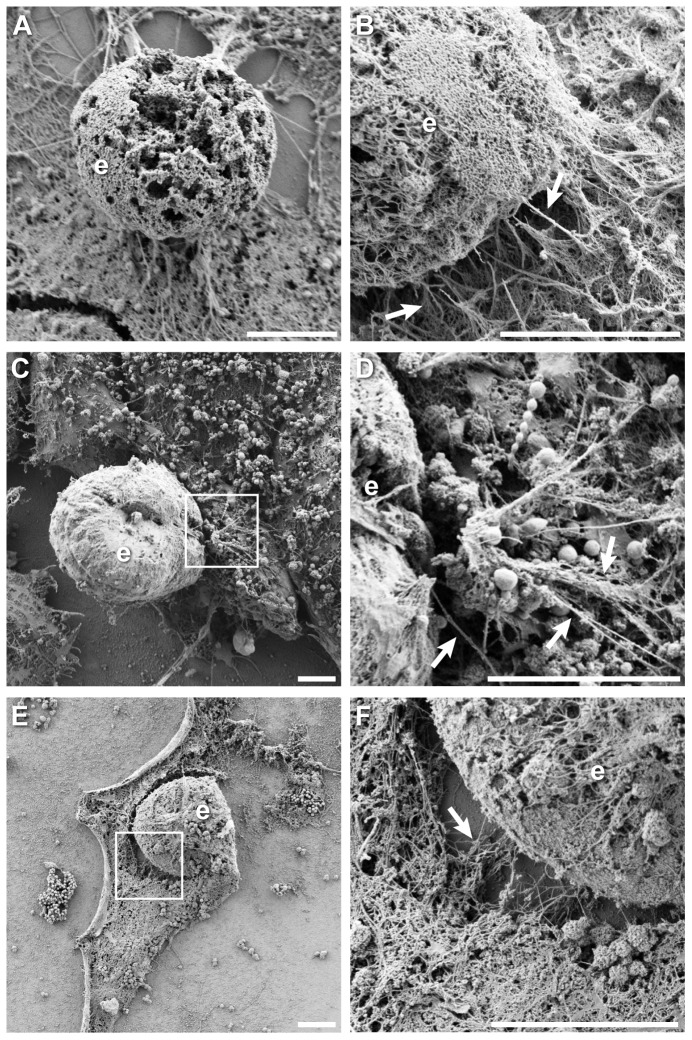
Ultrastructure of actin structures associated with *Chlamydia* extrusions. Images depict late stage inclusions and extrusions from cells infected with *C. trachomatis* at 68 hpi; in all instances specimens were processed by detergent extraction to reveal actin cytoskeleton. (**A**) A fractured extrusion, thrust vertically from the cell body. (**B**) Actin-coated extrusion detaching from the cytoskeleton of the cell body, some actin filaments remained bridging the extrusion and cellular actin matrix. (**C**) Extrusion with rich actin coat. (**D**) Enlargement of boxed region in (**C**), showing stretched actin bundles and partial detachment from cell body cytoskeleton. (**E**) Cell with large inclusion/extrusion. (**F**) Enlargement of boxed region in (**E**), showing zone of severed filaments between the extrusion/inclusion and actin network of the cell body. Extrusions are marked with *e*, some examples of actin filaments (arrows) are also labeled. Scale bars = 5 µm.

Finally, the unique characteristics of actin associated with *Chlamydia* inclusions immediately prior to and during the extrusion of bacteria from host cells was investigated. This warranted a closer look because of the potential paradox raised by our findings—how are extrusions ultimately released from cells if they are encased with a dense actin network that is continuous with the cytoskeleton of the cell body? We hypothesized that additional actin remodeling would be necessary in order to allow release of nascent extrusions from host cells. Detergent extracted SEM data were reexamined for cells that exhibited clear instances of extrusion. Representative examples are shown in Figure 9, for example the vertically-projected extrusion in Figure 9A. This analysis revealed that extrusions/inclusions frequently became detached from the cytoskeletal matrix of the cell body, and zones of severed filaments were frequently found around the vacuole (Figure 9B–F). This phenotype was only observed for late infected cells (68 hpi). Although we cannot entirely rule it out, these areas of separation did not appear to be an artifactual result of sample handling and processing; for example, some actin filaments remained bridging the space between vacuole and cell body (Figure 9B,F).

Another common observation of actin ultrastructure on nascent extrusions was the presence of long actin fibers or bundles that connected extrusions to the cellular matrix of actin (Figure 9A,B,D and Figure 2I–J). These actin cables may be actively involved with providing propulsive forces to extrusions, to facilitate their ejection out of the host cell. Alternatively they may be the indirect result of the extrusion—and its associated actin coat—stretching away from the cell cytoskeleton.

## Discussion

Late stage processes in *Chlamydia*-infected cells are poorly understood, particularly regarding the molecular mechanisms of interaction between the bacteria and host cells. The extrusion pathway of *Chlamydia* exit from host cells is emblematic of this. Although it has been established that extrusion requires co-option of actin polymerization and actin-associated signaling proteins by *Chlamydia* [10], the full molecular details of this orchestration remain undiscovered. Elucidation of these mechanisms are of fundamental importance to our understanding of the biology of chlamydiae, and also how these bacteria disseminate within a host and transmit to hew hosts.

Here, using a combination of real-time imaging of GFP labeled actin, immunofluorescence microscopy and SEM, we describe the detailed manner in which host-derived actin complexes are recruited and polymerized to the cytosolic surface of the *Chlamydia* inclusion. This event increased in both the extent of actin deposition (i.e., on a given inclusion) and prevalence throughout a cell monolayer over the chlamydial developmental cycle—from 20–68 hpi for *C. trachomatis* LGV, in agreement with the literature [24]. Moreover, extrusions exhibited the highest amount of actin coats, and de novo formation of extrusions was dependent on actin coat assembly. These findings are consistent with the established role of actin polymerization dynamics in mediating *Chlamydia* extrusion [10].

### Features and timing of actin recruitment to Chlamydia inclusions

Actin recruitment to the *Chlamydia* inclusion consisted of discrete steps. At 20 hpi, very little actin association with inclusions was detected. The only meaningful associations we observed were actin clouds in close proximity to the inclusion and puncta on the cytosolic face of the inclusion. The origin of these foci, and why they are first manifest in this manner (i.e., uncoupled from the inclusion membrane), are not clear; however, we propose that these are actin nucleation sites induced by *Chlamydia* type III secreted proteins.

As infection proceeds to 44 hpi, actin recruitment dramatically progressed to form arc (when viewed in 2-dimensionally) and partial cage structures (in 3-dimensions) on the regions of inclusion membranes distal to the nucleus. This particular spatial distribution was curious because it did not correlate with the location of 20 hpi actin clouds—which were typically adjacent to nuclei. At the ultrastructural level, actin deposition on inclusions at 44 hpi consisted of a rich layered actin network that was continuous with the actin matrix of the host cell.

At 68 hpi, and especially for extrusions, actin recruitment was much fuller, more deeply layered and frequently enwrapped the entire inclusion. When not completely circumscribing the inclusion, the regions of strongest actin recruitment were areas of the inclusion distal to the nucleus and where the inclusion protruded or bulged out of the swollen cell. We therefore speculate that these enriched areas of actin may mark the locations for imminent extrusions.

Actin recruitment to the *Chlamydia* inclusion is markedly different than what has been reported for other intracellular bacteria and pathogenic vacuoles, for example *Listeria*, *Rickettsia* spp., *Burkholderia pseudomallei* and *Mycobacterium marinum* [48], [49]. These bacteria employ strategies to initiate *y*-branched actin polymerization via the Arp2/3 complex and promote intracellular motility. Actin polymerization on these bacteria is rapid and appears as so-called flashes in live cells. Transient flashes of actin polymerization have also been demonstrated on the surface of parasitophorous vacuoles containing *Cryptococcus*, using a Arp2/3 and WASP based mechanism [26]. Actin structures on *Chlamydia* inclusions were dynamic yet did not exhibit rapid flash kinetics. Short term perturbation of host and bacterial signaling pathways in *Chlamydia* infected cells was sufficient to remove actin coats from inclusions, thus implicating their need for dynamic maintenance.

### Molecular mechanism

Resolution of the molecular pathways that give rise to this phenomenon are expected to inform new insights on how intracellular *Chlamydia* interact with host signaling pathways in order to promote infection, growth and survival. The mechanism of actin coat formation required cooption of host signaling pathways, most notably formins and septins. The strong role of formins argues for a filamentous nature of actin assembly, and this notion was corroborated by the presence of filamentous structures by SEM and the lack of an involvement of the Arp2/3 complex and N-WASP—which promote *y*-branched actin polymerization.

The involvement of Src family kinases in actin recruitment is particularly intriguing in the context of recent data showing the existence of microdomains of active SFK on *Chlamydia* inclusions that interact with four Inc proteins and centrosomes [16], [30]. These microdomains appear to be key foci of interactions between *Chlamydia* and host proteins, and play important roles in coordinating a number of signaling pathways in the cytosol: trafficking of nascent inclusions to the MTOC and processes that regulate *C. trachomatis* growth. Some *Chlamydia* species, for example *C. muridarum*, do not display active SFK microdomains on their inclusion surface and also do not associate with the MTOC [16], [30]. Also consistent with this theme is the involvement of microtubules in the formation of actin coats at 44 hpi. These results together suggest that interactions with the MTOC may be important for actin recruitment to the inclusion.

Myosin, Rho GTPase and ROCK function are all essential for extrusion formation [10], yet only myosin was involved in actin coat formation on inclusions. Much of these data are consistent with previous reports [24], yet there are some differences concerning the participation of Rho GTPase signaling. The reason for this discrepancy is unclear, although it might partly be attributable to the times hpi used for analysis, differences in MOI, or the overall heterogeneity of the actin recruitment phenotype.

### Septins

The participation of septins was unexpected, and represents the first description of a role for septins in *Chlamydia* pathogenesis. Septins are a conserved family of GTP-binding proteins that form nonpolar filamentous structures and associate with cellular membranes and the actin and microtubule cytoskeletons [41], [42], [50]. Septins have a wide range of functions in cells, such as cell division, membrane remodeling, cytoskeletal dynamics and acting as scaffolds for cytoskeleton and membrane proteins. Intriguingly, the structural integrity of septin and actin networks are intimately linked—depolymerization of actin filaments leads to a loss of septin fibers, and vice versa [45], [51], [52]. Septin polymerization has also been shown to be associated with Rho GTPase signaling [53]. In this context, we hypothesize that similar roles are fulfilled by septins during recruitment of actin to the *Chlamydia* inclusion, and that they may serve as a bridge between actin filaments and bacteria-derived proteins on the inclusion membrane. We additionally investigated the spatial distribution of the prominent septin Sept2 during *Chlamydia* infection; however, Sept2 distribution was not changed significantly with respect to infection or the *Chlamydia* inclusion (not shown).

### Chlamydial type III secretion products drive the event

Perhaps the salient finding of this work was the central role of *Chlamydia* type III secreted proteins in actively controlling actin coat assembly, and that extrusion was also dependent on chlamydial type III secreted product(s). We do not think Tarp is involved with this process, since Tarp staining did not colocalize with actin rings or foci, nor was Tarp found in the cytosol of cells at 20–68 hpi (not shown). Although their identity is unknown, the chlamydial proteins responsible exhibited a rapid turnover inside the host cell—as little as 1–2 hour treatment with chloramphenicol or rifampicin resulted in complete loss of actin-coated inclusions. Similar results were obtained by short-term treatment of cells with the C1 type III secretion inhibitor compound, which has been shown to effectively disrupt chlamydial type III secretion [46], [47], [54]. These temporal dynamics closely mirrored those for disruption of host signaling pathways, and therefore point to a mechanism of chlamydial and host proteins working in concert.

### Functional implications

Two emergent themes of this study are the striking heterogeneity of the phenomenon from cell-to-cell and the functional connection between actin coats and extrusion formation. Both of these themes inform new insight into *Chlamydia* biology and their interactions with the host cell.

Actin coat formation exhibited little to no partiality—inclusions either exhibited it fully (for their respective time of infection) or not at all, and this contrast was frequently observed for neighboring cells in a field. The simplest interpretation of this is that not all inclusions are modified equivalently and concurrently by *Chlamydia*. This could be due to: (*i*) differential timing of *Chlamydia* effector secretion into the host cytosol or onto the inclusion membrane, (*ii*) threshold levels of effector proteins that need to be met in balance with cognate host factors, (*iii*) differences among inclusions in the location of key secreted chlamydial proteins on the inclusion or in relation to cellular organelles. The subcellular distribution of actin coats showed clear patterns of organization—perinuclear actin foci for 20 hpi inclusions and arcs distal to nuclei for 44 hpi inclusions. These data are consistent with a spatial bias for actin nucleation on the inclusion. Moreover, there is a precedent for heterogeneous modifications of the *Chlamydia* inclusion by Inc proteins and their colocalization with Src family kinase microdomains [16], [30]. It is possible that the location of microdomain formation in relation to organelles and other cellular structures is critical for enabling their interaction with differential downstream signaling pathways in the host cell.

Finally, our data demonstrates that actin coating of inclusions is a key determining factor for extrusion; disruption of coat formation led to a marked reduction in the number of extrusions produced. This process may be functionally similar to a proposed mechanism for exocytosis of large secretory vesicles and organelles [55]. In addition, the majority of extrusions possessed strongly visible actin rings on their inclusions. The findings of the current study are highly consistent with previous data for the actin-based molecular mechanisms of *Chlamydia* extrusion [10]. Interestingly, the ∼50% prevalence of actin coated inclusions late in the developmental cycle closely mirrors the ∼50% frequency of extrusion *in vitro*. Both of these processes are directed by chlamydial proteins, and future identification of these proteins will reveal how intracellular *Chlamydia* induce their escape from host cells by the novel extrusion mechanism.

## Materials and Methods

### Reagents

Unless otherwise specified, all reagents and chemicals were obtained from Sigma (St. Louis, MO). Additional reagents and concentrations used include: chloramphenicol (100 µg/ml), rifampicin (100 µg/ml), C1 compound N′-(3,5-dibromo-2-hydroxybenzylidene)-4-nitrobenzohydrazide (50 µM) (ChemBridge, San Diego, CA), jasplakinolide (200 nM) (Invitrogen, Carlsbad, CA), latrunculin B (500 nM) (Invitrogen), CK-548 (10 µM) (Enzo Life Sciences, Ann Arbor, MI), CK-666 (10 µM) (EMD Millipore, Billerica, MA), blebbistatin (10 µM) (EMD Millipore), phalloidin (Invitrogen), wiskostatin (10 µM) (EMD Millipore), small molecule inhibitor of formin homology 2 domain (10 µM) (SMIFH2, EMD Millipore), LY294002 (50 µM) (Enzo), cell permeable C3 transferase CT04 (2.5 µg/ml) (Cytoskeleton, Denver, CO), Y27632 (20 µM) (Enzo), Src Kinase Inhibitor I (25 µM) (EMD Millipore), nocodazole (10 µM).

### Cell culture and infections

HeLa 229 and L929 cells [10], [56] and McCoy cells, kindly provided by Julius Schachter [57], were routinely grown in RPMI 1640 media supplemented with 10% FBS (HyClone, Thermo Fisher Scientific, Rockford, IL) and 2 mM L-glutamine (HyClone), at 37°C with CO_2_. For all experiments, cells were subcultured and plated onto chambered coverglass slides (Lab-Tek II; Nunc, Rochester, NY), glass bottom culture dishes (MatTek, Ashland, MA) or 24-well plates (BD Falcon).

*Chlamydia trachomatis* serovars L2 and D, and *C. muridarum* were grown for 48 h in L929 cells as described previously [57]. Chlamydial EB were isolated by sonic disruption of L929 suspensions and purification by centrifugation. EB were resuspended in sucrose-phosphate-glutamic acid buffer and stored in aliquots at −80°C.

Infections were performed by washing cells with Hank's balanced saline solution (HBSS, HyClone) and incubating cells with *Chlamydia* EB diluted in HBSS to a multiplicity of infection ≤1, for 2 h at 25°C. Following static incubation, cells were rinsed twice with HBSS, reimmersed in growth media and incubated at 37°C.

### Transfections

Cells with transient expression of cytosolic DsRed and LifeAct-GFP were generated by co-transfecting freshly plated cells with 0.1 µg each of DsRed-Express (Clontech) and LifeAct–TagGFP2 (Ibidi, Munich, Germany). This was routinely performed 6 hours prior to infection with *Chlamydia*, and transfections were performed using the Effectene reagent (Qiagen), per manufacturer's protocol.

### Enumeration of actin recruitment to Chlamydia inclusions

Experiments using infected, DsRed- and LifeAct-GFP-expressing cells were performed in triplicate or wells of 4-well or 8-well chambered coverglass slides. At times hpi indicated, cells were washed with RPMI without phenol red and mounted live on a Zeiss Axiovert 200 inverted microscope with a Orca-R2 cooled digital CCD camera (Hamamatsu, Japan), 40× oil objective (1.3NA Plan-NEOFLUAR) and Sedat Quad filter set (Chroma, Bellows Falls, VT). Three *z*-slices were acquired for each cell or field, using a *z*-focus motor drive (Ludl, Hawthorne, NY) under control of Volocity Acquisition software v6 (PerkinElmer, Shelton, CT) on an Apple iMac (Apple, Cupertino, CA). Fields of cells were selected at random and imaged if they contained identifiable inclusions by DsRed fluorescence [10]; red and green color channels were imaged. Depending on the cell density, 10–20 fields per well were acquired. *Chlamydia* inclusions were scored as positive for actin recruitment if any of the *z*-slices for a particular cell exhibited significant association of fluorescent actin on the inclusion; the minimum criteria for significance was an arc of actin that circumscribed at least ∼30% of the inclusion boundary.

### Fluorescence videomicroscopy

Infected cells for real-time imaging were grown and prepared in 4-well chambered coverglass slides, and rinsed and incubated with serum-free RPMI media lacking phenol red (HyClone) prior to mounting on the microscope. Slide chambers were secured and imaged on a Nikon TE2000-E inverted microscope (Nikon, Tokyo, Japan), with a CoolSnap HQ cooled CCD camera (Photometrics, Tucson, AZ), 40× oil objective (1.3NA Plan Fluor), with computer control of an *xy* stage (Applied Scientific Instrumentation, Eugene, OR) and *z* focus (PerfectFocus, Nikon), a humidified incubation chamber for maintaining environmental control of 37°C temperature and 5% CO_2_ (In Vivo Scientific), and using NIS elements software (Nikon). Images were captured every 5 minutes for 6–8 hours. Time-lapse recordings were exported into Volocity for further analysis, and movies were compiled using Volocity and QuickTime (Apple).

### Detergent extraction scanning electron microscopy

Cells were grown to subconfluency on 10 mm square silicon wafers (Ted Pella, Redding, CA) and infected with *C. trachomatis* as described above. Samples undergoing detergent extraction were rinsed with PBS and incubated for 5 min at 25°C in an extraction solution consisting of 1% Triton X-100, 2% PEG 35 kD and 2 µM phalloidin that was prepared in 1X PEM buffer: 100 mM PIPES, pH 6.9, 1 mM MgCl_2_, 1 mM EGTA. All samples were then rinsed three times with PEM and fixed with 2.5% glutaraldehyde (Electron Microscopy Sciences, Hatfield, PA) for at least 20 minutes at 25°C. Samples were stored in fixative at 4°C until ready for further processing using techniques described [27]. Briefly, fixed samples were immersed in a 0.1% tannic acid solution for 20 minutes at room temperature. The samples were then rinsed in distilled water and immersed in 0.2% aqueous uranyl acetate solution for 20 minutes. Next, the samples were dehydrated using an ethanol gradient (10, 20, 40, 60, 80, 100% ethanol) for 5 minutes each. Afterwards, the samples were soaked in 0.2% uranyl acetate in ethanol for 20 minutes. After two rinses in 100% ethanol for 5 minutes each, the samples were critical point dried using a Samdri®-795 critical point dryer (Tousimis Research Corporation, Rockville, MD). Afterwards, the samples were coated with iridium for conductivity and imaged using a Zeiss Supra 55VP field emission scanning electron microscope with 1 kV accelerating voltage.

### Post-acquisition image processing

In some instances, three dimensional image stacks were further processed in Volocity by performing illumination correction (in *z* dimension) and deconvolution (25 iterations). Individual *xy* and *xz* slices were obtained from image stacks for figure assembly. Three dimensional opacity renderings of fluorescent image stacks were generated in Volocity. Minor retouching of all micrographs—for example, color assignment, contrast adjustment, RGB merges and cropping—were performed with Volocity and Photoshop CS5 (Adobe). Illustrator CS5 (Adobe) was used to assemble all figures into their final form: add scale bars, arrows, labels, etc.

### Extrusion isolation

At 68 hpi, infected cells were aspirated, given fresh growth media and allowed to incubate at 37°C for 4 hours. Culture supernatants containing extrusions were harvested and cleared of cell debris by centrifugation (5 min at 75× g). Extrusions were subsequently enriched by centrifugation (10 min at 1200 rpm); pellets containing extrusions were resuspended in 1–5 mL growth media. To enumerate the number of extrusions obtained from a cell monolayer, resuspended extrusions were stained with SYTOX Green (1∶2000, Molecular Probes) and Hoechst (1∶2000, Molecular Probes) for 5 minutes at 25°C, plated as 5–10 µl drops onto glass slides and imaged immediately on an inverted fluorescence microscope. Intact extrusions were identified as having chlamydial inclusions, lacking nuclei and being the appropriate size.

### Statistical analysis

Statistical evaluation of data was performed by calculating the standard error of the mean (SEM) or using a two-tailed student *t*-test. P-values <0.01 (*) were considered statistically significant. P-values of <0.001 (**) and <0.0001 (***) were marked as indicated. Calculations were performed in Numbers (Apple) and Microsoft Excel.

## Supporting Information

Figure S1 **Three dimensional distribution of LifeAct-GFP in** ***Chlamydia*** **infected cells.** HeLa cells expressing LifeAct-GFP (green) and cytosolic DsRed (magenta) were infected with *C. trachomatis* L2 and imaged at 20 hpi (**A**), 44 hpi (**B**) and 68 hpi (**C**) by live microscopy. Over 55 z-stacks were acquired at 0.5 µm intervals. Image stacks were processed by deconvolution. Representative fields of cells for each time point are depicted. The cell in (**B**) and the leftmost cell in (**C**) were also rendered in three dimensions to show the extensiveness of actin coating on the inclusion. In each case, views were rotated slightly to yield the most informative perspective. Insets in (**A**) are magnifications of the inclusion in the lower left cell. Asterisks (*) mark the locations where orthogonal planes in *xz* were taken. Arrows in (**A**) mark the inclusions. Scale bars = 20 µm.
(TIF)

Figure S2 **Three dimensional distribution of endogenous actin filaments in** ***Chlamydia*** **infected cells.** HeLa cells were infected with *C. trachomatis* L2, and fixed and processed for immunofluorescence at 20 hpi (**A**), 44 hpi (**B**) and 68 hpi (**C**). Immunofluorescence staining was performed using phalloidin conjugated to Alexa633 (purple), a specific antibody to *Chlamydia* MOMP (green) and the nucleic acid dye DAPI (blue) for staining nuclei and, to a lesser extent, bacteria. The partial staining of nuclei and bacteria in the actin channel are likely artifact, and due to bleedthrough of Evans blue dye (red, not shown). A total of 17, 28 and 56 *z*-stacks were acquired at 0.5 µm intervals for (**A**), (**B**) and (**C**), respectively. Image stacks were processed by deconvolution. Representative fields of cells for each time point are depicted. Asterisks (*) mark the locations where orthogonal planes in *xz* were taken. Arrows in (**A**) and (**B**) mark the inclusions. Scale bars = 20 µm.
(TIF)

Video S1 **Three dimensional rendering of GFP-actin recruitment to a** ***C. trachomatis*** **L2 inclusion at 48 hpi.** Cell is labeled with LifeAct-TagGFP (green) and cytosolic DsRed (magenta). Three dimensional rendering and rotation are from the cell depicted in Figure S1 (B).
(MOV)

Video S2 **Three dimensional rendering of GFP-actin recruitment to a** ***C. trachomatis*** **L2 inclusion at 72 hpi.** Cell is labeled with LifeAct-TagGFP (green) and cytosolic DsRed (magenta). Three dimensional rendering and rotation are from the leftmost cell depicted in Figure S1 (C).
(MOV)

Video S3 **Time-lapse videomicroscopy of GFP-actin in** ***C. trachomatis*** **L2 infected cell at 48 hpi.** Cell is labeled with LifeAct-TagGFP (green) and cytosolic DsRed (magenta). Frames were captured every 5 minutes.
(MOV)

Video S4 **Time-lapse videomicroscopy of GFP-actin in** ***C. trachomatis*** **L2 infected cell at 24 hpi.** Cell is labeled with LifeAct-TagGFP (green) and cytosolic DsRed (magenta). Frames were captured every 5 minutes.
(MOV)
