# Supplementary figures and images for: Actin Recruitment to the Chlamydia Inclusion Is Spatiotemporally Regulated by a Mechanism That Requires Host and Bacterial Factors
Source: PLoS One. 2012 Oct 11;7(10):e46949. doi: 10.1371/journal.pone.0046949 (PMC3469565; doi:10.1371/journal.pone.0046949)

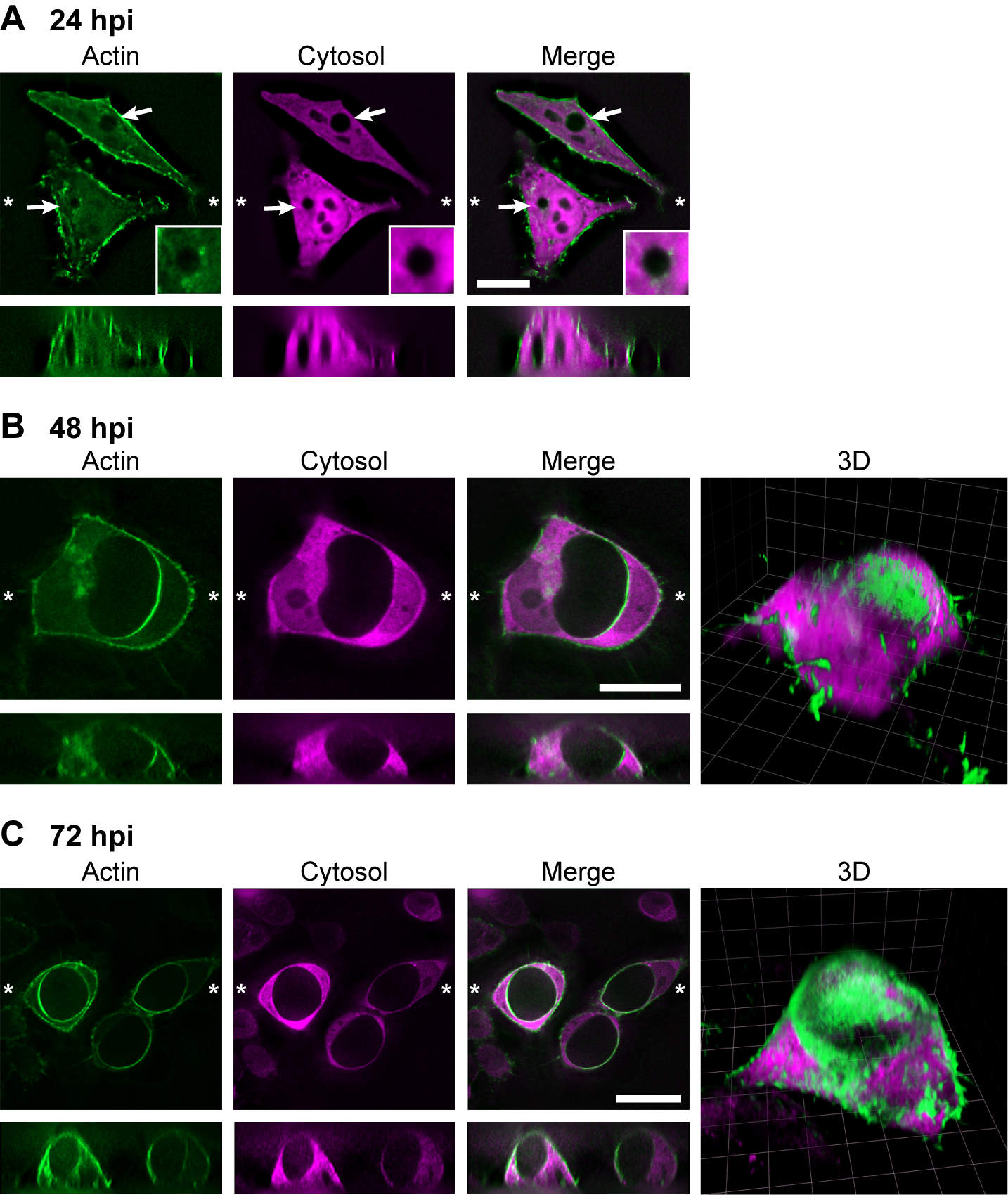

Supplement: Figure S1 — Three dimensional distribution of LifeAct-GFP in Chlamydia infected cells. HeLa cells expressing LifeAct-GFP (green) and cytosolic DsRed (magenta) were infected with C. trachomatis L2 and imaged at 20 hpi (A), 44 hpi (B) and 68 hpi (C) by live microscopy. Over 55 z-stacks were acquired at 0.5 µm intervals. Image stacks were processed by deconvolution. Representative fields of cells for each time point are depicted. The cell in (B) and the leftmost cell in (C) were also rendered in three dimensions to show the extensiveness of actin coating on the inclusion. In each case, views were rotated slightly to yield the most informative perspective. Insets in (A) are magnifications of the inclusion in the lower left cell. Asterisks (*) mark the locations where orthogonal planes in xz were taken. Arrows in (A) mark the inclusions. Scale bars = 20 µm. (TIF) [file pone.0046949.s001.tif]

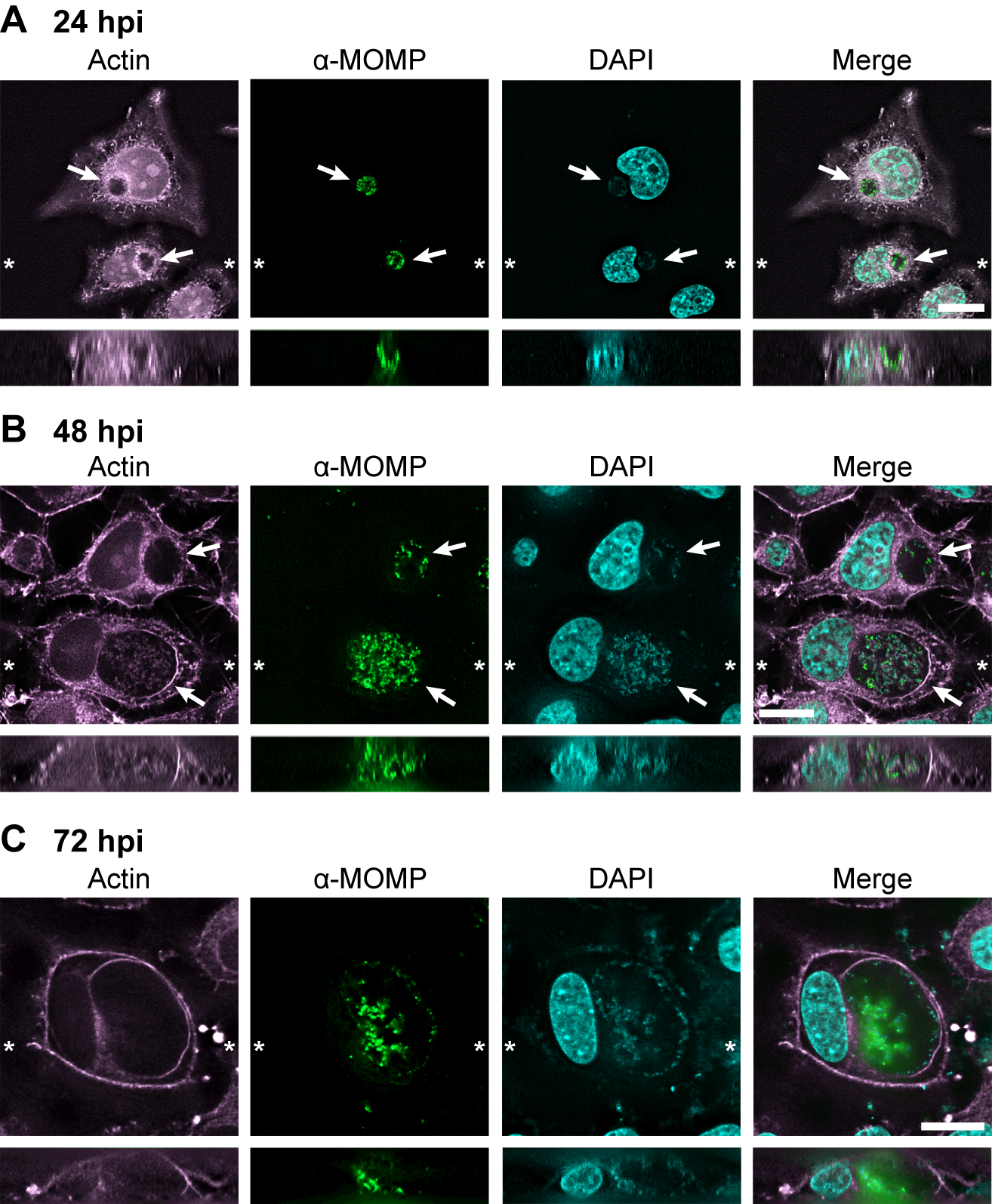

Supplement: Figure S2 — Three dimensional distribution of endogenous actin filaments in Chlamydia infected cells. HeLa cells were infected with C. trachomatis L2, and fixed and processed for immunofluorescence at 20 hpi (A), 44 hpi (B) and 68 hpi (C). Immunofluorescence staining was performed using phalloidin conjugated to Alexa633 (purple), a specific antibody to Chlamydia MOMP (green) and the nucleic acid dye DAPI (blue) for staining nuclei and, to a lesser extent, bacteria. The partial staining of nuclei and bacteria in the actin channel are likely artifact, and due to bleedthrough of Evans blue dye (red, not shown). A total of 17, 28 and 56 z-stacks were acquired at 0.5 µm intervals for (A), (B) and (C), respectively. Image stacks were processed by deconvolution. Representative fields of cells for each time point are depicted. Asterisks (*) mark the locations where orthogonal planes in xz were taken. Arrows in (A) and (B) mark the inclusions. Scale bars = 20 µm. (TIF) [file pone.0046949.s002.tif]
